# Neurological mechanisms of mental fatigue in cognitive flexibility in soccer players: an event-related potential study based on N2 components

**DOI:** 10.3389/fnins.2025.1729495

**Published:** 2025-12-05

**Authors:** Chen Gong, Jun Zhao, Yifan Wang, Xuejian Ding

**Affiliations:** 1 College of Physical Education, Northeast Electric Power University, Jilin, China; 2 Brain Science Institute, Jilin Medical University, Jilin, China

**Keywords:** mental fatigue, soccer players, cognitive flexibility, event-related potential, neurological mechanisms

## Abstract

**Objectives:**

Mental fatigue frequently occurs in team sports characterised by high cognitive demands and can adversely affect athletes’ decision-making and executive functions. This study examines the impact of mental fatigue on cognitive flexibility in soccer players, employing event-related potentials (ERP) to elucidate the underlying neural mechanisms.

**Method:**

Eighteen football players from the China University Football Association (CUFA) participated in the study. The research utilised the Stroop task to induce mental fatigue and the More-odd switching task to evaluate cognitive flexibility, with measurement indicators comprising accuracy rate (ACC), reaction time (RT), and N2 component amplitude. The experimental design adhered to a repeated measures protocol, incorporating 2 (time: pre-fatigue/post-fatigue) × 2 (task type: conversion/non-conversion) × 4 (electrode positions: Fz, Cz, Pz, F3) factors. Statistical analyses of behavioural data were conducted using non-parametric tests, while ERP data were examined through repeated measures ANOVA.

**Results:**

Mental fatigue significantly impaired athletes’ accuracy in the More-odd switching task, evidenced by decreased performance in both conversion and non-conversion conditions (*p* < 0.05), alongside prolonged reaction times (*p* < 0.01). Event-related potential (ERP) analysis demonstrated a marked reduction in the amplitudes of the N2 component post-intervention, with notable differences across electrode sites (*F* = 3.192, *p* = 0.031). These findings suggest a restriction in cognitive control resources within the frontal and parietal regions.

**Conclusion:**

This study illustrates that experimentally induced mental fatigue has a significant detrimental impact on the cognitive performance of football players, leading to slower reaction times and decreased neural electrophysiological indicators (specifically, a reduction in N2 wave amplitude), indicating a decline in conflict monitoring ability. An integrated examination of both behavioural and neurophysiological data indicates that the adverse effects of mental fatigue are likely due to the depletion of general cognitive control resources that rely on the prefrontal cortex, rather than targeting particular cognitive functions. These results offer initial insights into the cognitive and neurological alterations in fatigued athletes. While the study did not explore interventions, the findings lay the groundwork for the development of tailored cognitive training programmes and fatigue management strategies, along with potential evaluation criteria. Notably, the N2 wave amplitude serves as a key metric for assessing cognitive resource levels in football players.

## 1 Introduction

Mental fatigue is defined as a psychobiological state resulting from prolonged and intense cognitive demands (Boksem and Tops, 2008). It is characterised by subjective sensations of “fatigue” and “lack of energy” (Rozand and Lepers, 2017). This condition is prevalent in team sports that require significant cognitive engagement and operate within unpredictable environments (Yuan et al., 2023). Research indicates that mental fatigue adversely affects both physical and tactical performance in soccer players (Coutinho et al., 2017). The cognitive challenges inherent in team sports such as soccer are particularly formidable (Faubert and Sidebottom, 2012). For example, during matches, the movements of both teammates and opponents are unpredictable, and the trajectory of the ball cannot be anticipated; distractions may necessitate changes in direction, while the movements of others can obstruct the path of the incoming ball (Smith et al., 2018). These overwhelming streams of information hinder decision-making capabilities and ultimately impact athletic performance (Skala and Zemková, 2022). Consequently, athletes must sustain a high level of cognitive flexibility throughout the duration of the match (Sun et al., 2021).

The essence of football resides in the intense confrontations and counterattacks between opposing teams and their players, as both sides endeavour to outmanoeuvre one another. Beyond the physical demands inherent to the sport, players consistently encounter the challenge of being marked by opponents. In such high-pressure scenarios, they must make decisions that benefit their team (Trecroci et al., 2020). Given the multifaceted performance requirements in modern football, cognition may be the pivotal factor in achieving and sustaining peak performance (Knöbel and Lautenbach, 2023). With the evolution of modern football, advanced tactical concepts such as high pressing and structured defence have emerged, imposing extreme demands on players’ physical endurance (Sally, 2014). Consequently, the time and space available to players on the field have significantly diminished (Vestberg et al., 2020). In this context, executive functions—namely inhibition, cognitive flexibility, and working memory—are regarded as essential for effective and goal-oriented behaviour (Diamond, 2013).

Recent studies have highlighted an increasing interest in the relationship between mental fatigue and football performance, particularly its effects on physical conditioning (Smith et al., 2015; Coutinho et al., 2018; Filipas et al., 2020), technical skills (Smith et al., 2016), tactical execution (Kunrath et al., 2020), cognitive functions (Hans-Erik and Daniel, 2019), and training methodologies (Bian et al., 2022). For example, mental fatigue significantly influences players’ offensive and defensive techniques, notably diminishing both the frequency and success rate of tackles (Sun et al., 2022). Another investigation revealed that, although mental fatigue does not compromise athletes’ maximal strength, explosive power, or anaerobic capacity (Van Cutsem et al., 2017), its interaction with motivation during endurance training can affect performance (Dantzer et al., 2014). Moreover, mental fatigue exacerbates the sensation of “I cannot do it” by activating inhibitory neural systems in the brain. Cognitive research concerning football players has largely concentrated on developing assessment tools (Kane et al., 2007) and analysing athletes’ inhibitory contro (Soylu et al., 2022), decision-making abilities (Fortes et al., 2020), working memory (Zhou, 2021), and visual skills (Knöllner et al., 2022). However, further investigation is warranted to understand how mental fatigue impacts cognitive flexibility. Cognitive flexibility refers to the ability to consciously adjust cognitive strategies in response to environmental changes, enabling individuals to adapt to new situations and solve novel problems. Essentially, it involves switching between different cognitive rules, reflecting an individual’s capacity for mental transformation and inhibitory control (Zühlsdorff et al., 2023).

The Wisconsin Card Sorting Test (WCST) and the task-switching paradigm are currently prevalent methods for assessing cognitive flexibility (Schmitter-Edgecombe and Langill, 2006; Nyhus and Barceló, 2009). Nevertheless, these approaches predominantly concentrate on behavioural data from athletes, with limited investigation into the underlying neural mechanisms. Prior research has demonstrated a correlation between working memory, cognitive flexibility, and goal-scoring performance throughout a player’s season. This relationship remains significant even when controlling for intelligence, height, and age in partial correlation analyses (Wallace and Norton, 2014). Studies suggest that high executive function can serve as a predictor of football success among young players. While earlier investigations have explored the influence of cognitive functions in elite versus novice athletes and across various age groups through EEG mapping, no research has yet validated the mechanisms by which mental fatigue impacts cognitive flexibility in football players, particularly from the perspective of the More-odd shifting task (Dong et al., 2022). EEG spectral measurements offer a comprehensive overview of rhythmic brain activity, whereas event-related potentials (ERP) are temporally confined to specific events or stimuli, enabling a more precise examination of the neural processes that underpin cognitive functions (Sokhadze et al., 2017).

Classical event-related potential (ERP) components encompass P1, N1, P2, N2, and P3. The early ERP components, namely P1, N1, and P2, are regarded as exogenous factors predominantly influenced by the physical properties of stimuli (Luck and Kappenman, 2012). In contrast, the later-emerging components, such as N2 and P3, are considered endogenous factors closely linked to individual cognitive processes, including attention, decision-making, and memory updating (Folstein and Van Petten, 2008). The N2 component, which appears prior to P3, is characterised by a negative wave occurring approximately 200 milliseconds after stimulus onset, reflecting the brain’s initial processing of stimuli (Fan et al., 2023). Research suggests that goal-directed behaviour relies on control mechanisms associated with the prefrontal cortex, and task-switching processes can be employed to investigate cognitive flexibility mechanisms (Rozand and Lepers, 2017). In the context of EEG methodologies, switching mechanisms are represented by the N2 and P3 ERP components; during task switching, the N2 ERP component indicates mechanisms involved in resolving conflicts between simultaneously active stimulus–response mappings (Gehring et al., 2003). Other studies have reported increased N2 amplitudes following prolonged tasks (Boksem et al., 2005; Möckel et al., 2015). Additionally, research focusing on elderly individuals and patients has demonstrated that heightened N2 amplitudes signify increased energy costs associated with cognitive control, suggesting that patients utilise more resources in response selection (Pinal et al., 2015).

Building upon existing ERP literature concerning cognitive control and task switching (Trecroci et al., 2020), this study selected four electrode sites—Fz, Cz, Pz, and F3—for analysis. Firstly, Fz and Cz are situated in the precentral scalp region, representing the most prominent areas for conflict-related N2 components, which effectively reflect conflict monitoring activity in the anterior cingulate cortex (ACC) (Folstein and Van Petten, 2008). Secondly, the F3 site is located above the dorsal anterior frontal lobe (DLPFC), a region closely linked to task set reconfiguration and executive control, thereby facilitating a deeper exploration of the specific neural mechanisms underlying cognitive flexibility (Thönes et al., 2018). Finally, the Pz site records activity from the parietal cortex, serving dual purposes: confirming the anterior distribution characteristics of N2 components and preparing for subsequent stimulus evaluation analysis (Kieffaber and Hetrick, 2005). In this study, we not only focused on the N2 components associated with conflict monitoring recorded by midline electrodes (Fz, Cz, Pz), but also specifically established the F3 electrode in the left prefrontal cortex. The F3 electrode, located at the standard position of the international 10–20 system, is widely recognised for its ability to effectively reflect the neural activity of the underlying left dorsolateral prefrontal cortex (Ota et al., 2019).

Mental fatigue, as a critical factor influencing cognitive flexibility, necessitates a thorough examination of its relationship with cognitive performance. Comprehending this connection is vital for devising targeted interventions aimed at enhancing athletes’ capacity to manage complex situations and improve their cognitive adaptability. Consequently, this study synthesises existing evidence regarding the impact of mental fatigue on cognitive flexibility in soccer players, while also investigating the neural mechanisms that underpin this relationship.

## 2 Methods

### 2.1 Participants

An *a priori* power analysis was conducted using G*Power software (version 3.1) (Faul et al., 2009). The analysis selected the *t*-test for means: difference between two dependent means (matched pairs)model to evaluate differences in ERP components before and after the mental fatigue intervention. Based on findings from established literature (Boksem et al., 2005), which consistently report large to very large effects of mental fatigue on ERP components such as P3 and N2 (with reported Cohen’s d > 0.8) (Kato et al., 2009), a conservatively large effect size (d = 0.8) was set for this study. For a two-tailed test with an alpha level of 0.05 and a statistical power of 80%, the analysis indicated a required sample size of *N* = 15 participants. Accounting for potential data loss due to signal artefacts (approximately 10%) and the specific characteristics of the participant population, a total of *N* = 18 soccer players were recruited. The final sample had a mean age of 20.5 ± 1.2 years and a mean training experience of 8.5 ± 2.5 years. All participants were right-handed.

The inclusion criteria were as follows: (1) No use of insomnia medications or similar drugs; (2) Participants had to be professional football players; (3) No history of concussions; and (4) No significant differences were found in the two rounds of International Physical Activity Questionnaire (IPAQ) assessments. Exclusion criteria included: (1) History of chronic or major illnesses; (2) Sports-related injuries or fractures within the past 3 years; and (3) Participation in high-intensity activities (e.g., basketball, running, skiing) within 48 h prior to testing. All participants signed written informed consent forms. This study was approved by the Ethics Committee of Northeast Electric Power University (No. 2024-1-822).

### 2.2 Procedure

This experiment utilised a mixed experimental design comprising 2 (time: pre-fatigue, post-fatigue) × 2 (task type: switching tasks, non-switching tasks) × electrode positions (FZ, CZ, PZ, F3). The behavioural task results were analysed using accuracy rate and reaction time as dependent variables, with time functioning as a group-level variable. Behavioural measurements were obtained through the More-odd shifting task, where accuracy rate (ACC) and reaction time (RT) served as key metrics. Electroencephalographic measurements employed event-related potentials (ERP), with a specific focus on the amplitude of the N2 component. All procedures were executed using E-prime 3. software, and cognitive flexibility was assessed via the More-odd switching task. The intervention experiment was conducted employing the Stroop paradigm for stimulus induction. The Stroop and More-odd conversion tasks employed in this study are classic paradigms for measuring cognitive control and task-switching, with their reliability and validity well-established in extensive neuroscience and psychology research (Stroop, 1935; Miyake et al., 2000; Schmit and Brisswalter, 2018). Notably, these tasks have been successfully applied to cognitive assessments in athletes and ERP studies (Verburgh et al., 2014; Sun et al., 2022), demonstrating their validity in similar experimental contexts.

### 2.3 Measures

**Figure 1:**
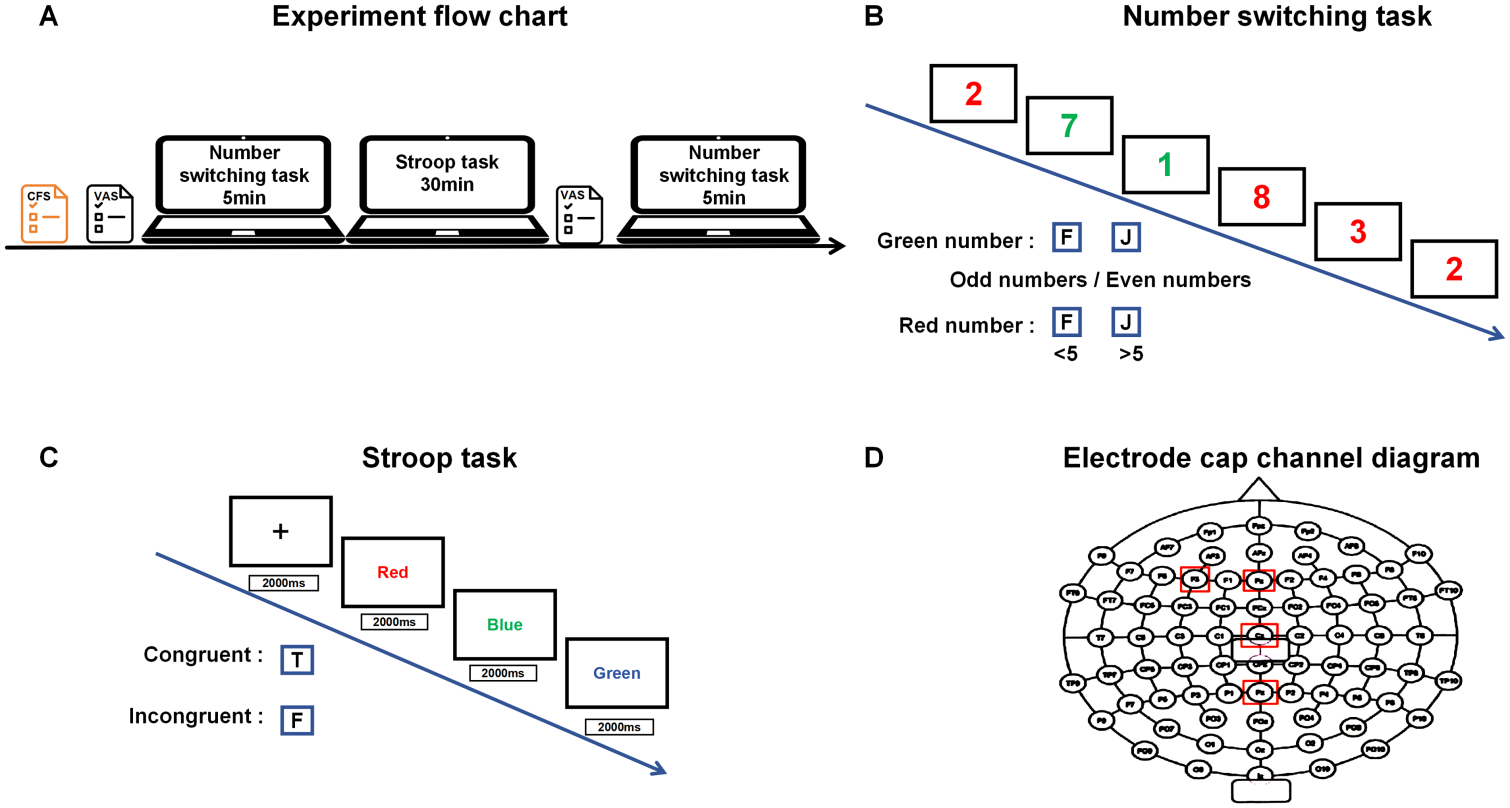
Schematic diagram of the experimental procedure and experimental paradigm.

As shown in Figure 1, all participants completed the Cognitive Flexibility Scale (CFS) test prior to the experiment to assess their baseline executive function levels45. The Cognitive Flexibility Scale (CFI), originally developed by Dennis and Vander Wal (2010), was later adapted into a Chinese version by Wang et al. (2016). This 20-item scale measures two dimensions (substitution and control), with example items such as “I am good at analysing and evaluating various situations.” The scale demonstrated a Cronbach’s alpha coefficient of 0.847. Results indicated that all participants maintained normal executive function levels, with no significant differences observed between groups (*p* > 0.05). This finding effectively ruled out baseline differences from influencing experimental outcomes. Subsequently, a 30-min Stroop task (Pinal et al., 2015) was administered to induce mental fatigue. After confirming participants’ fatigue status through subjective assessment, a modified odd-even switching task was employed to evaluate cognitive flexibility.

#### 2.3.1 Mental fatigue task

**Figure 2:**
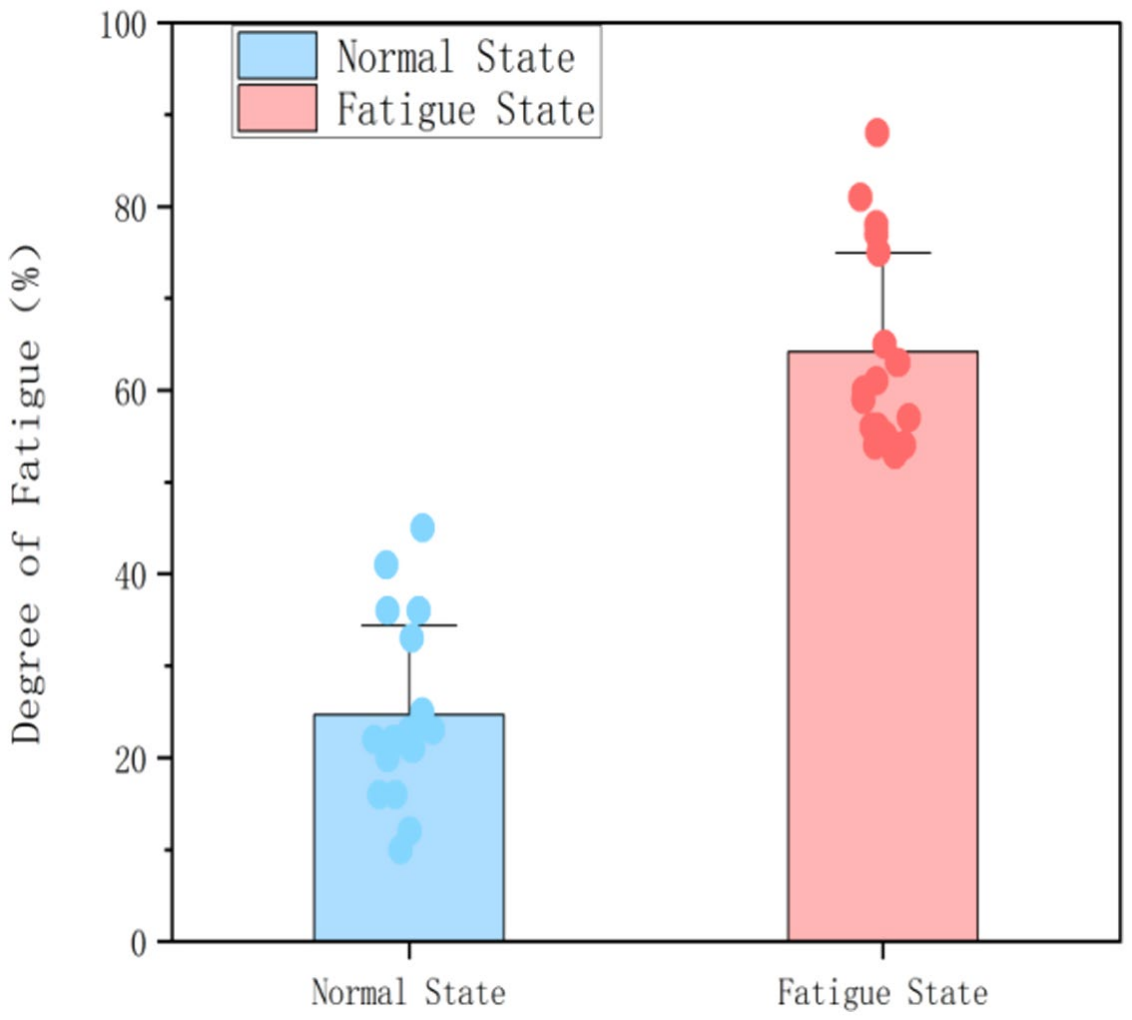
Difference test of Visual Analog Scale (VAS) scores before and after Stroop task.

Prior research has established the Stroop task as a reliable experimental model for inducing mental fatigue (Meymandi et al., 2023). Participants engage in a cognitively demanding process that necessitates the suppression of automatic responses, which progressively results in mental fatigue. The 30-min duration employed in this study was adapted from effective mental fatigue induction methods utilised in similar previous studies (Niu et al., 2024). In accordance with Catala et al. (2021), the implementation of our Stroop task adhered to the principles outlined below (as detailed in Figure 2).

#### 2.3.2 Subjective mental fatigue evaluation

Participants’ subjective levels of mental fatigue were evaluated using the Visual Analog Scale (VAS), a specific instrument developed for measuring mental fatigue, as noted in previous research (Smith et al., 2019). The VAS consists of a 100-millimetre line with endpoints designated as mm (“no mental fatigue”) and 100 mm (“maximum mental fatigue”). Participants indicated their perceived levels of fatigue by marking points along this scale.

Participants were instructed to assess their mental fatigue using a 10-point scale, with the left end denoting “no mental fatigue” and the right end signifying “complete mental exhaustion.” They indicated their subjective experiences along this continuum. The Visual Analog Scale (VAS) was employed on two occasions: first as a baseline measure, and subsequently, one minute after the completion of the Stroop task. A score greater than 50 was deemed indicative of considerable mental fatigue, as established in the study by Meymandi et al. (2023).

#### 2.3.3 More-odd switching task

**Figure 3:**
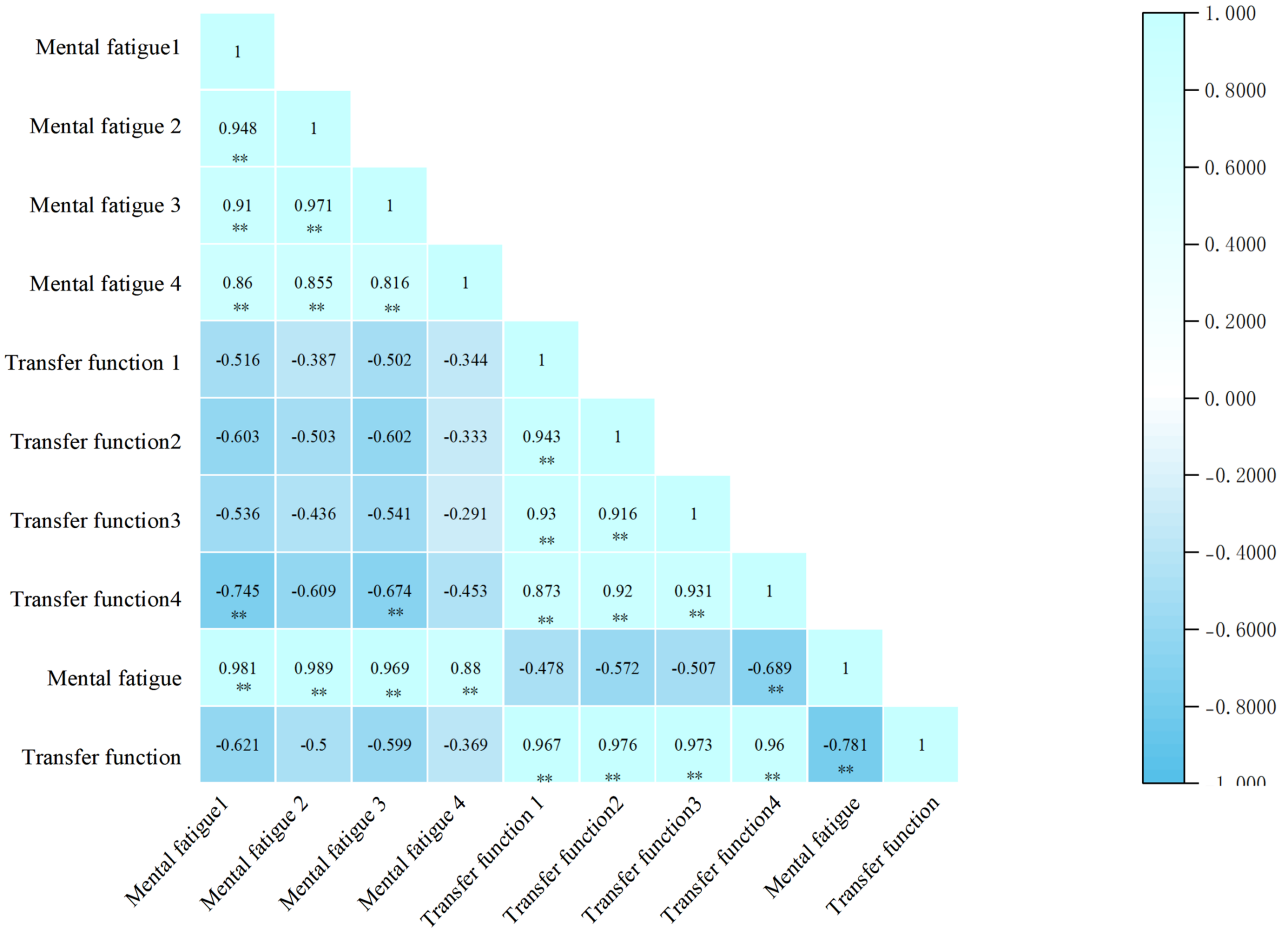
Heat map of the correlation between mental fatigue and cognitive flexibility.

The formal experiment for the odd-number switching task consists of three distinct phases. Phase One requires participants to view a green number, assess its parity, and subsequently press the corresponding key: “F” for odd numbers and “J” for even numbers. In Phase Two, participants will observe a red number, evaluate its magnitude, and then press “F” for values less than 5 or “J” for those greater than 5. This phase functions as a non-switching trial. Phase Three integrates both previous phases into a combined switch trial (Hillman et al., 2006). The design and procedure of the odd-number switching task are depicted in Figure 3. After implementing the preprocessing pipeline described above, we quantified the number of valid trials retained for each participant to ensure the reliability of the ERP averaging process. For the Stroop task, an average of 102 valid trials per participant (range: 88 to 114 trials) were retained, resulting in an average trial retention rate of 85%. For the More-odd shifting task, an average of 128 valid trials per participant (range: 110 to 142 trials) were retained, with an average retention rate of 80%. The minimum number of trials retained for any participant in any condition exceeded 40, which is well above the commonly accepted threshold of 30 trials for obtaining stable ERP components (Olvet and Hajcak, 2009), thereby ensuring the robustness of our subsequent ERP analyses.

#### 2.3.4 EEG recording, preprocessing and ERP

**Figure 4:**
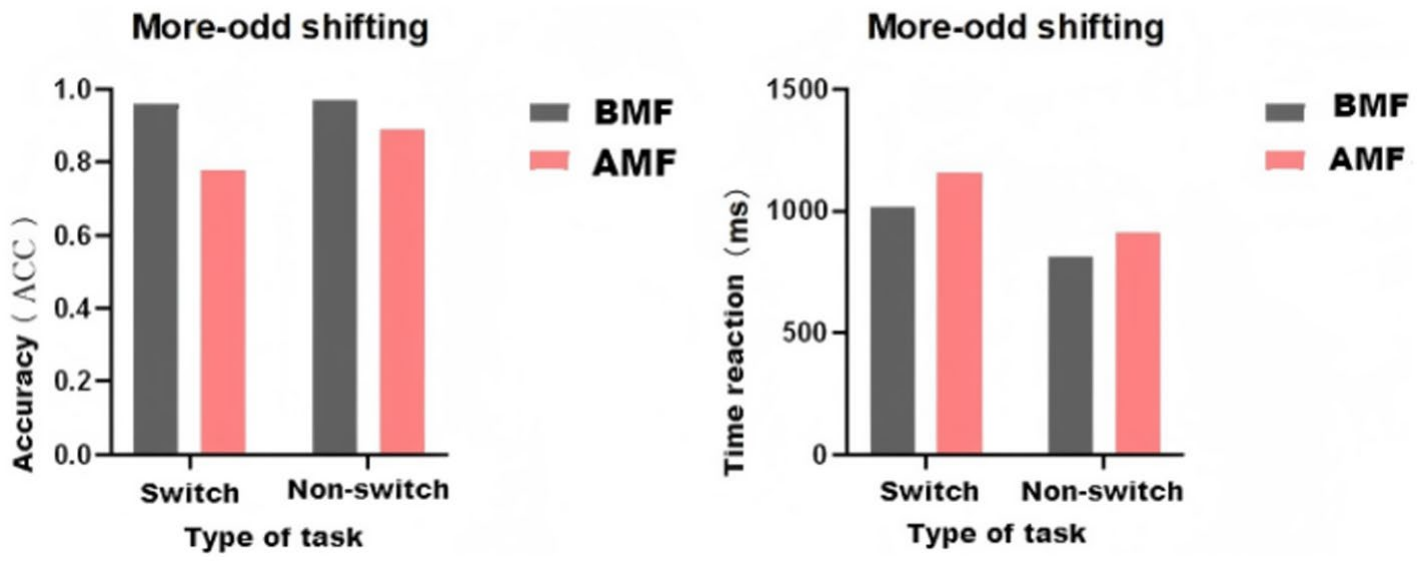
Histogram of test results for accuracy and reaction time in the More-odd shifting task before and after mental fatigue (BMF: before mental fatigue; AMF: after mental fatigue).

Electroencephalogram (EEG) data were collected using a 32-channel head-mounted device (Emotiv FLEX EEG system, developed by Emotiv Systems, operating at 128 Hz), in accordance with the international 10–10 system guidelines. Prior to the study, participants underwent a standardised scalp cleansing procedure to minimise sebum interference and reduce electrode impedance. Electrolyte gel was applied to each electrode position to ensure optimal contact between the electrodes and the scalp, thereby maintaining impedance levels below 10 kΩ. EEG recordings were obtained at a sampling rate of 500 Hz, with online filtering configured to 0.1–0.100 Hz. The configuration of the electrode channels is depicted in Figure 4.

The EEG data underwent a series of preprocessing steps aimed at enhancing signal quality (Niu et al., 2024). This preprocessing was conducted using the EEGLab toolbox within the Matlab 2023b environment. The processing workflow is outlined as follows: Initially, the raw data were subjected to bandpass filtering within the range of 0.1–0.40 Hz. During data acquisition, the reference electrode was positioned at FCz. In the preprocessing phase, the data were re-referenced to the average values of the bilateral mastoid electrodes (TP9/TP10) as the offline reference. Subsequently, all data were combined and analysed using independent component analysis (ICA) to identify and eliminate artefact components associated with blinking, eye movements, and electromyographic activity, thereby reconstructing clean EEG data.

(1) Behavioural Data: The behavioural data were merged and preprocessed using E-Merge. Instances of trials with erroneous responses were excluded, followed by the removal of abnormal trials in which reaction times were either shorter than 100 ms or longer than 200 ms within correct trials. Statistical analyses of behavioural metrics, specifically accuracy rate and reaction time, were conducted using SPSS 26. software, with a significance level set at *α* = 0.05.
(2) EEG Data: Recording and Analysis EEG data were collected using the Emotiv FLEX system and subsequently analysed offline within the Matlab 2023b environment. The preprocessing workflow commenced with re-referencing the data to a whole-brain average reference. This was followed by the application of band-pass filtering within the range of 0.1 to 30 Hz. Independent Component Analysis (ICA) was employed to semi-automatically identify and remove eye movement artefacts. Brainwave epochs were recorded from 200 ms prior to stimulus presentation to 800 ms following it, with baseline calibration conducted using the period from 200 ms before to ms prior to the stimuli. Trials deemed invalid, characterised by amplitudes exceeding ±100 μV, were automatically excluded.

Drawing upon the overall average T-wave waveform and referencing previous literature (Kopp et al., 1996), the analysis time window for N2 components in the More-odd shifting task was established as 150–200 ms (Smith et al., 2016). The average amplitudes from four electrode sites (Fz, Cz, Pz, F3) within this time window were extracted and subjected to statistical analysis using SPSS 26. For the Stroop task, we compared pre-and post-intervention differences in “congruent reaction time”, “incongruent reaction time”, and “Stroop interference effect (incongruent-congruent)” between mental fatigue groups. As all differences followed a normal distribution (*p* > 0.05), we conducted paired *t*-tests for analysis.

For the More-odd conversion task, we compared mental fatigue intervention effects on conversion cost (conversion trial reaction time minus repetition trial reaction time) and average accuracy. The conversion cost data showed non-normal distribution (*p* < 0.05), so we used Wilcoxon’s sign-rank test—a nonparametric test—for analysis. All tests were conducted with a *p* < 0.05 significance threshold.

## 3 Results

### 3.1 Nonparametric test results for the More-odd shifting task under neural fatigue intervention

As shown in Table 1, the accuracy rates of conversion and non-conversion responses (*p* = 0.013, *p* = 0.032) and response times (*p* = 0.026, *p* = 0.100, *p* = 0.026, *p* = 0.026) before and after mental fatigue did not follow a normal distribution. Therefore, the rank sum test was applied. As shown in Table 2, the rank-sum test revealed statistically significant differences in the accuracy and reaction time of conversion and non-conversion responses before and after mental fatigue. Specifically, the conversion response accuracy (Z = −2.1, *p* = 0.036) and reaction time (Z = −1.9, *p* = 0.048) showed statistically significant differences, while the non-conversion response accuracy (Z = −3.8, *p* = 0.015) and reaction time (Z = −4.2, *p* = 0.028) also demonstrated statistically significant differences.

**Table 1:** Normality test for the More-odd shifting task.

	Time	Type of test	Statistic	*df*	*p*
Accuracy	Pre-mental fatigue	Switch	0.152	32	0.013
		Non-switch	0.342	32	0.018
	Post-mental fatigue	Switch	0.391	32	0.032
		Non-switch	0.511	32	0.000
Reaction Time	Pre-mental fatigue	Switch	0.923	32	0.026
		Non-switch	0.882	32	0.002
	Post-mental fatigue	Switch	0.944	32	0.100
		Non-switch	0.923	32	0.026

**Table 2:** Wilcoxon signed-rank test for the More-odd shifting task.

		Pre-mental fatigue	Post-mental fatigue	Statistics	
		M (p25, p75)		*Z*	*p*
Accuracy	Switch	90% (85, 94%)	87% (82, 91%)	−2.21	0.036*
	Non-switch	95% (92, 98%)	93% (89, 96%)	−1.90	0.048*
Reaction time	Switch	810 (720, 950)	890 (770, 1,050)	−3.80	0.015*
	Non-switch	680 (600, 790)	750 (650, 880)	−4.20	0.028*

*indicates *p* < 0.05.

### 3.2 Results of difference test before and after mental fatigue

**Figure 5:**
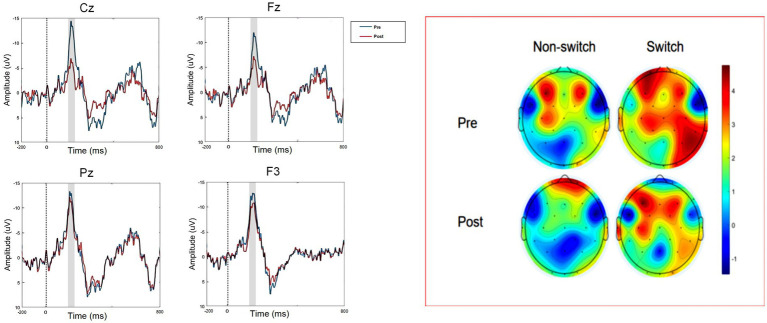
Changes in potential waveforms and topographic maps at each electrode site before and after mental fatigue.

Figure 5 illustrates that the paired-sample *t*-test identified a significant difference in Visual Analog Scale (VAS) scores before and after the Stroop task (*t* = −13.436, *p* < 0.001). The pre-task average VAS score was 24.667 ± 9.726, while the post-task average score was 64.166 ± 10.798, indicating that the 30-min Stroop task effectively induced mental fatigue in participants.

### 3.3 Results of correlation analysis between mental fatigue and cognitive flexibility in football players

Illustrates a notable negative correlation between mental fatigue, measured at electrode points Fz, Cz, Pz, and F3, and cognitive flexibility, assessed at the same electrode points (r = −0.781, *p* < 0.01). This finding suggests that as participants’ levels of mental fatigue increase, their cognitive flexibility decreases.

### 3.4 Results of the correct rate of More-odd shifting task under mental fatigue intervention

Table 3 illustrate that, in conversion tasks, the correct response rate for the More-odd shifting task prior to the mental fatigue intervention was (0.96 ± 0.17)%, whereas subsequent to the intervention, it decreased to (0.78 ± 0.42)%. In non-conversion tasks, the correct response rate before the intervention was (0.97 ± 0.16)%, which subsequently dropped to (0.89 ± 0.31)%. These results indicate that participants exhibited a decline in the correct response rate for the More-odd shifting task following mental fatigue, thereby suggesting a reduction in conversion ability within this cognitive domain. The repeated measures ANOVA results revealed a significant main effect of time [*F*(1, 17) = 2.953, *p* = 0.012, η^2^p = 0.148], while the main effect of trial type was not significant [*F*(1, 17) = 0.660, *p* = 0.418, *η^2^p* = 0.037]. Additionally, the time × trial type interaction was not significant [*F*(1, 17) = 0.165, *p* = 0.685, *η^2^p* = 0.010].

**Table 3:** ANOVA of repeated measurements of accuracy under the More-odd shift task.

	Before mental fatigue	After mental fatigue	Repeat the F-test		
	M ± SD	M ± SD	*F*	*P*	*η^2^p*
Switch	0.96 ± 0.17	0.78 ± 0.42			
NO-switch	0.97 ± 0.16	0.89 ± 0.31			
Time			2.953	0.012	0.148
Type of test			0.660	0.418	0.037
Time × type of test			0.165	0.685	0.010

### 3.5 Reaction time results of More-odd shifting task under mental fatigue intervention

Table 4 illustrate that during conversion trials, the reaction time for the More-odd shifting task was (1017.069 ± 426.924/ms) prior to the mental fatigue intervention, whereas it increased to (1158.098 ± 494.587/ms) following the intervention. In non-conversion trials, the response time remained at (811.608 ± 377.291/ms) pre-intervention but rose to (911.670 ± 419.075/ms) post-intervention. Analysis of variance (ANOVA) results indicated significant main effects of time [*F*(1, 17) = 5.638, *p* = 0.030, *η^2^p* = 0.249] and trial type [*F*(1, 17) = 3.998, *p* = 0.042, *η^2^p* = 0.0190]. while no significant interaction was observed between time and trial type [*F*(1, 17) = 3.746, *p* = 0.070, *η^2^p* = 0.181].

**Table 4:** ANOVA of repeated measures of reaction time under the More-odd shifting task.

	Before mental fatigue (ms)	After mental fatigue (ms)	Repeat the F-test		
	M ± SD	M ± SD	*F*	*P*	*η^2^p*
Switch	1017.06 ± 426.92	1158.0 ± 494.58			
NO-switch	811.60 ± 377.29	911.67 ± 419.07			
Time			5.638	0.030	0.249
Type of test			3.988	0.042	0.190
Time × type of test			3.746	0.070	0.181

### 3.6 Electroencephalographic measurements of the More-odd shifting task under cerebral fatigue intervention

To examine the alterations in cognitive flexibility preceding and following mental fatigue, we utilised the latency of the N2 component from the More-odd shifting task as the dependent variable. The amplitude of the event-related potential N2 component was assessed through repeated measurements of differences (RMD) employing a two-way ANOVA design that incorporated three factors: time (mental fatigue before versus after), trial type (transition trials versus non-transition trials), and electrode positions (FZ, CZ, PZ, F3).

The examination of Tables 5, 6 reveals that mental fatigue (time factor) and brain regions (electrode point factor) exert a significant influence on the N2 latency period, while the task type and its interactions with other factors do not show significant effects. The main effect of time was significant [*F*(1, 17) = 8.401, *p* = 0.010, *η^2^p* = 0.331], revealing considerable variations in N2 amplitude across different time intervals. Similarly, the main effect of electrode point was significant [*F*(3, 51) = 3.192, *p* = 0.031, *η^2^p* = 0.158], indicating notable differences in N2 latency across various brain regions (different electrode recording points). The interaction effect between time and electrode point was also significant [*F*(3, 51) = 2.854, *p* = 0.041, *η^2^p* = 0.144], suggesting that the influence of mental fatigue on N2 latency differs among brain regions. In contrast, the main effect of task type was not significant [*F*(1, 17) = 0.662, *p* = 0.423, *η^2^p* = 0.037], implying that, after controlling for fatigue and time factors, different types of odd-even conversion tasks did not yield significant differences in N2 latency. All interactions involving task type were non-significant: time × task type (*p* = 0.572), electrode point × task type (*p* = 0.415), and the third-order interaction electrode point × time × task type (*p* = 0.529) showed no significant effects. This suggests that the manner in which mental fatigue influences N2 latency is consistent across various task types, indicating that task type does not moderate the relationship between fatigue and brain responses.

**Table 5:** Latency of N2 component under mental fatigue intervention in More-odd shift task.

	Before mental fatigue (M ± SD)		After mental fatigue (M ± SD)	
	Switch	NO-switch	Switch	NO-switch
CZ	13.360 ± 1.034	12.699 ± 0.801	3.104 ± 3.654	2.515 ± 5.843
FZ	14.055 ± 1.073	13.902 ± 1.861	4.444 ± 3.461	3.834 ± 5.025
PZ	18.346 ± 1.487	18.542 ± 2.680	6.133 ± 1.773	5.199 ± 1.407
F3	23.744 ± 0.742	24.642 ± 1.460	16.766 ± 3.433	17.018 ± 4.169

**Table 6:** Repeated measures ANOVA of N2 component latency under More odd shift tasks.

	df	*F*	*P*	*η^2^p*
Electrode points	3	3.192	0.031	0.158
Time	1	8.401	0.010	0.331
Type of test	1	0.662	0.423	0.037
Time × type of test	1	0.324	0.572	0.018
Time× electrode points	3	2.854	0.041	0.144
Electrode points × test type	3	0.951	0.415	0.053
Electrode points × time × test type	3	0.753	0.529	0.042

## 4 Discussion

This study provides robust evidence that a 30-min Stroop task effectively induces mental fatigue in soccer players, as demonstrated by significant subjective reports, prolonged reaction times, and decreased accuracy. Notably, our neurophysiological data reveal that this behavioural impairment is underpinned by a specific neural mechanism: a marked reduction in N2 amplitude and an increased latency during the More-odd shifting task (Liu et al., 2025), particularly observable at the Cz electrode site. The synthesis of these behavioural and electrophysiological findings allows us to propose a coherent model: mental fatigue compromises athletic cognitive performance primarily by depleting the neural resources essential for early conflict monitoring and cognitive control (Norman and Bobrow, 1975), as indicated by the diminished N2 component, which directly leads to slower and less accurate responses.

A Coherent Neuro-Behavioural Model of Fatigue-Induced Impairment The observed decline in behavioural performance aligns with the well-established concept of finite attentional resources (Hancock and Desmond, 2001). Our study extends this principle by identifying a plausible neural correlate. The N2 component, generated in the anterior cingulate cortex (ACC) and dorsolateral prefrontal cortex (DLPFC), is a well-established neural marker of conflict detection and cognitive control (Folstein and Van Petten, 2008; Nieuwenhuis et al., 2003). The significant reduction in N2 amplitude following fatigue indicates a less efficient neural response in these key prefrontal regions (Petruo et al., 2018). This means that the soccer players’ brains became less adept at rapidly detecting and signalling the cognitive conflict inherent in the task-switching paradigm. This “neural inefficiency” at the early stage of information processing (~200–300 ms) forces subsequent cognitive processes to operate on a degraded signal, ultimately manifesting as the delayed reaction times and increased errors we recorded. Therefore, the N2 attenuation is not merely an parallel observation; it is the mechanistic link that explains why behaviour deteriorates under fatigue—the core computational machinery for cognitive control is compromised (Sarter et al., 2006).

A critical analysis of the existing literature and the theoretical implications of our study reveal a significant discovery. We found a consistent reduction in N2 amplitude during both switch and non-switch trials, suggesting that mental fatigue impacts a broad cognitive control mechanism rather than just the switching process. This observation provides empirical backing for the Limited Cognitive Control Theory (Hockey, 2013; Baumeister et al., 1998), which posits that prolonged cognitive exertion depletes a central pool of cognitive resources (Ishii et al., 2014).

When placed in the wider context of existing literature, our findings demonstrate both similarities and differences that enrich the field. The discovery of compromised cognitive adaptability under fatigue corresponds with various studies in sports (Smith et al., 2018; Sokhadze et al., 2017). Nonetheless, the particular decrease in N2 amplitude we have identified offers a more intricate viewpoint. Previous research has indicated an increase in N2 among non-athletes or in different fatigue scenarios, suggesting heightened neural exertion (Lorist et al., 2005). In contrast, our distinct reduction in N2 may be elucidated by participant characteristics and task specificity. Elite footballers, accustomed to streamlined and automatic cognitive processes, may operate near their neural capacity thresholds. Consequently, when fatigue diminishes their resources, their capacity for neural compensation may be rapidly depleted, resulting in a reduced neural reaction rather than the heightened effort seen in other groups. This highlights that the neural effects of fatigue are not uniform but are shaped by individual diversities and task requirements (Tran et al., 2020).

It is crucial to translate these findings into practical applications to bridge the theory-practise gap. Our research suggests that cognitive performance, a key factor in soccer success, is vulnerable to mental fatigue (Smith et al., 2016). Therefore, practitioners should consider the following strategies: Firstly, coaches should manage cognitive load during training sessions similar to physical load periodization. Sessions with high tactical and cognitive demands should be followed by sufficient recovery or lighter cognitive activities to prevent cumulative fatigue that could impede learning and decision-making (Schmit and Brisswalter, 2018). Secondly, athletes should avoid mentally fatiguing activities (prolonged studying, intense video gaming, stressful work) in the 24 to 48 h before a competition to maintain the sharp cognitive control needed during the game. Additionally, athletes could benefit from “cognitive reset” techniques such as brief breathing exercises or temporarily shifting focus during halftime or breaks to replenish cognitive resources and potentially improve performance in the second half (Nédélec et al., 2015). Lastly, training programmes should include specific exercises to enhance cognitive endurance and efficiency under fatigue, strengthening compromised neural systems (as indicated by the N2) (Nédélec et al., 2013; Van der Linden et al., 2003).

## 5 Conclusion

This study illustrates that experimentally induced mental fatigue has a significant detrimental impact on the cognitive performance of football players, leading to slower reaction times and decreased neural electrophysiological indicators (specifically, a reduction in N2 wave amplitude), indicating a decline in conflict monitoring ability. An integrated examination of both behavioural and neurophysiological data indicates that the adverse effects of mental fatigue are likely due to the depletion of general cognitive control resources that rely on the prefrontal cortex, rather than targeting particular cognitive functions. These results offer initial insights into the cognitive and neurological alterations in fatigued athletes. While the study did not explore interventions, the findings lay the groundwork for the development of tailored cognitive training programmes and fatigue management strategies, along with potential evaluation criteria. Notably, the N2 wave amplitude serves as a key metric for assessing cognitive resource levels in football players.

### 5.1 Limitations and future prospects

A key limitation of this study is the use of a single-group pre-post design without a control group that performed neutral tasks while undergoing identical measurements. Although we have minimised potential confounding factors through experimental design, this limitation may still compromise the internal validity of the results. Future research should incorporate well-designed control groups to more rigorously isolate the specific neurophysiological effects of mental fatigue, while further exploring the potential impacts of different types of control tasks.

Although this study preliminarily revealed the negative impact of mental fatigue on cognitive flexibility of soccer players by combining behavioural and neurophysiological indicators, there are still some limitations: small sample size: this study only included 18 college soccer players, and the limited sample size may affect the stability of statistical inference and the extrapolation of results.

High homogeneity of participants: All participants were from the same college football association (CUFA), with similar training background and competitive level, which limited the applicability of the research conclusions to athletes at different levels.

Single way of inducing fatigue: only Stroop task was used to induce mental fatigue, without football-specific cognitive tasks or real game situation, which has limited support for ecological validity.

## Data Availability

The raw data supporting the conclusions of this article will be made available by the authors, without undue reservation.

## References

[ref1] Baumeister R. F. Bratslavsky E. Muraven M. Tice D. M. (1998). Ego depletion: is the active self a limited resource? J. Pers. Soc. Psychol. 74, 1252–1265. doi: 10.1037/0022-3514.74.5.1252, 9599441

[ref2] Bian C. Ali A. Nassis G. P. Li Y. (2022). Repeated interval Loughborough soccer passing tests: an ecologically valid motor task to induce mental fatigue in soccer. Front. Physiol. 12:803528. doi: 10.3389/fphys.2021.803528, 35126183 PMC8811352

[ref3] Boksem M. A. Meijman T. F. Lorist M. M. (2005). Effects of mental fatigue on attention: an ERP study. Cogn. Brain Res. 25, 107–116. doi: 10.1016/j.cogbrainres.2005.04.011, 15913965

[ref4] Boksem M. A. Tops M. (2008). Mental fatigue: costs and benefits. Brain Res. Rev. 59, 125–139. doi: 10.1016/j.brainresrev.2008.07.001, 18652844

[ref5] Catala P. Gutierrez L. Écija C. Serrano Del Moral Á. Peñacoba C. (2021). Do cognitive abilities influence physical and mental fatigue in patients with chronic pain after walking according to a clinical guideline for physical exercise? Int. J. Environ. Res. Public Health 18:13148. doi: 10.3390/ijerph182413148, 34948758 PMC8701060

[ref6] Coutinho D. Gonçalves B. Travassos B. Wong D. P. Coutts A. J. Sampaio J. E. (2017). Mental fatigue and spatial references impair soccer players' physical and tactical performances. Front. Psychol. 8:283443. doi: 10.3389/fpsyg.2017.01645 PMC5613114 28983273

[ref7] Coutinho D. Gonçalves B. Wong D. P. Travassos B. Coutts A. J. Sampaio J. (2018). Exploring the effects of mental and muscular fatigue in soccer players’ performance. Hum. Mov. Sci. 58, 287–296. doi: 10.1016/j.humov.2018.03.004, 29549745

[ref8] Dantzer R. Heijnen C. J. Kavelaars A. Laye S. Capuron L. (2014). The neuroimmune basis of fatigue. Trends Neurosci. 37, 39–46. doi: 10.1016/j.tins.2013.10.003, 24239063 PMC3889707

[ref9] Dennis J. P. Vander Wal J. S. (2010). The cognitive flexibility inventory: instrument development and estimates of reliability and validity. Cogn. Ther. Res. 34, 241–253. doi: 10.1007/s10608-009-9276-4

[ref10] Diamond A. (2013). Executive functions. Annu. Rev. Psychol. 64, 135–168. doi: 10.1146/annurev-psych-113011-143750, 23020641 PMC4084861

[ref11] Dong Z. Wang P. Xin X. Li S. Wang J. Zhao J. . (2022). The relationship between physical activity and trait anxiety in college students: the mediating role of executive function. Front. Hum. Neurosci. 16:1009540. doi: 10.3389/fnhum.2022.1009540 36211122 PMC9540794

[ref12] Fan J. Li W. Lin M. Li X. Deng X. (2023). Effects of mindfulness and fatigue on emotional processing: an event-related potentials study. Front. Behav. Neurosci. 17:1175067. doi: 10.3389/fnbeh.2023.1175067, 37304761 PMC10249016

[ref13] Faubert J. Sidebottom L. (2012). Perceptual-cognitive training of athletes. J. Clin. Sport Psychol. 6, 85–102. doi: 10.1123/jcsp.6.1.85

[ref14] Faul F. Erdfelder E. Buchner A. Lang A. G. (2009). Statistical power analyses using G* power 3.1: tests for correlation and regression analyses. Behav. Res. Methods 41, 1149–1160. doi: 10.3758/BRM.41.4.1149, 19897823

[ref15] Filipas L. Borghi S. La Torre A. Smith M. R. (2020). Effects of mental fatigue on soccer-specific performance in young players. Sci. Med. Footb. 5, 150–157. doi: 10.1080/24733938.2020.1823012, 35077334

[ref16] Folstein J. R. Van Petten C. (2008). Influence of cognitive control and mismatch on the N2 component of the ERP: a review. Psychophysiology 45, 152–170. doi: 10.1111/j.1469-8986.2007.00602.x, 17850238 PMC2365910

[ref17] Fortes L. S. De Lima-Junior D. Fiorese L. Nascimento-Júnior J. R. A. Mortatti A. L. Ferreira M. E. C. (2020). The effect of smartphones and playing video games on decision-making in soccer players: a crossover and randomised study. J. Sports Sci. 38, 552–558. doi: 10.1080/02640414.2020.1715181, 31941416

[ref18] Gehring W. J. Bryck R. L. Jonides J. Albin R. L. Badre D. (2003). The mind's eye, looking inward? In search of executive control in internal attention shifting. Psychophysiology 40, 572–585. doi: 10.1111/1469-8986.00059, 14570165

[ref19] Hancock P. A. Desmond P. A. (2001). Stress, workload, and fatigue. Lawrence Erlbaum Associates Publishers. Available online at: 10.1002/hfm.1009 (Accessed June 18, 2024).

[ref20] Hans-Erik S. Daniel M. (2019). The relationship between cognitive functions and sport-specific motor skills in elite youth soccer players. Front. Psychol. 10:817. doi: 10.3389/fpsyg.2019.00817 31105611 PMC6494938

[ref21] Hillman C. H. Kramer A. F. Belopolsky A. V. Smith D. P. (2006). A cross-sectional examination of age and physical activity on performance and event-related brain potentials in a task switching paradigm. Int. J. Psychophysiol. 59, 30–39. doi: 10.1016/j.ijpsycho.2005.04.009, 16413382

[ref22] Hockey R. (2013). The psychology of fatigue: Work, effort and control. Cambridge University Press. Available online at: 10.1017/CBO9781139015394.

[ref23] Ishii A. Tanaka M. Watanabe Y. (2014). Neural mechanisms of mental fatigue. Rev. Neurosci. 25, 469–479. doi: 10.1515/revneuro-2014-0028, 24926625

[ref24] Kane M. J. Conway A. R. Miura T. K. Colflesh G. J. (2007). Working memory, attention control, and the N-back task: a question of construct validity. J. Exp. Psychol. Learn. Mem. Cogn. 33, 615–622. doi: 10.1037/0278-7393.33.3.615, 17470009

[ref25] Kato Y. Endo H. Kizuka T. (2009). Mental fatigue and impaired response processes: event-related brain potentials in a go/NoGo task. Int. J. Psychophysiol. 72, 204–211. doi: 10.1016/j.ijpsycho.2008.12.008, 19135100

[ref26] Kieffaber P. D. Hetrick W. P. (2005). Event-related potential correlates of task switching and switch costs. Psychophysiology 42, 56–71. doi: 10.1111/j.1469-8986.2005.00262.x, 15720581

[ref27] Knöbel S. Lautenbach F. (2023). An assist for cognitive diagnostics in soccer (part II): development and validation of a task to measure working memory in a soccer-specific setting. Front. Psychol. 13:1026017. doi: 10.3389/fpsyg.2022.1026017, 36817381 PMC9936861

[ref28] Knöllner A. Memmert D. von Lehe M. Jungilligens J. Scharfen H. E. (2022). Specific relations of visual skills and executive functions in elite soccer players. Front. Psychol. 13:960092. doi: 10.3389/fpsyg.2022.960092, 36092125 PMC9454603

[ref29] Kopp B. Rist F. Mattler U. (1996). N200 in the flanker task as a neurobehavioral tool for investigating executive control. Psychophysiology 33, 282–294. doi: 10.1111/j.1469-8986.1996.tb00425.x, 8936397

[ref30] Kunrath C. A. Cardoso F. D. S. L. Calvo T. G. Costa I. T. D. (2020). Mental fatigue in soccer: a systematic review. Rev. Bras. Med. Esporte 26, 172–178. doi: 10.1590/1517-869220202602208206

[ref31] Liu Q. Huang R. Liu Z. Sun C. Qi L. Cicchella A. (2025). The impact of mental fatigue on the accuracy of penalty kicks in college soccer players. Sports (Basel) 13:259. doi: 10.3390/sports13080259, 40863768 PMC12390445

[ref32] Lorist M. M. Boksem M. A. Ridderinkhof K. R. (2005). Impaired cognitive control and reduced cingulate activity during mental fatigue. Brain Res. Cogn. Brain Res. 24, 199–205. doi: 10.1016/j.cogbrainres.2005.01.018, 15993758

[ref33] Luck S. J. Kappenman E. S. (Eds.) (2012). The Oxford handbook of event-related potential components. New York, USA: Oxford University Press.

[ref34] Meymandi N. P. Sanjari M. A. Farsi A. (2023). The effect of mental and muscular fatigue on the accuracy and kinematics of dart throwing. Percept. Mot. Skills 130, 808–825. doi: 10.1177/00315125221146613, 36606603

[ref35] Miyake A. Friedman N. P. Emerson M. J. Witzki A. H. Howerter A. Wager T. D. (2000). The unity and diversity of executive functions and their contributions to complex "frontal lobe" tasks: a latent variable analysis. Cogn. Psychol. 41, 49–100. doi: 10.1006/cogp.1999.0734, 10945922

[ref36] Möckel T. Beste C. Wascher E. (2015). The effects of time on task in response selection—an ERP study of mental fatigue. Sci. Rep. 5:10113. doi: 10.1038/srep10113, 26054837 PMC4460573

[ref37] Nédélec M. Halson S. Abaidia A. E. Ahmaidi S. Dupont G. (2015). Stress, sleep and recovery in elite soccer: a critical review of the literature. Sports Med. 45, 1387–1400. doi: 10.1007/s40279-015-0358-z, 26206724

[ref38] Nédélec M. McCall A. Carling C. Legall F. Berthoin S. Dupont G. (2013). Recovery in soccer. Sports Med. 43, 9–22. doi: 10.1007/s40279-012-0002-0, 23315753

[ref39] Nieuwenhuis S. Yeung N. Van Den Wildenberg W. Ridderinkhof K. R. (2003). Electrophysiological correlates of anterior cingulate function in a go/no-go task: effects of response conflict and trial type frequency. Cogn. Affect. Behav. Neurosci. 3, 17–26. doi: 10.3758/CABN.3.1.17, 12822595

[ref40] Niu S. Guo J. Hanson N. J. Wang K. Chai J. Guo F. (2024). The effects of mental fatigue on fine motor performance in humans and its neural network connectivity mechanism: a dart throwing study. Cereb. Cortex 34:bhae085. doi: 10.1093/cercor/bhae085, 38489786

[ref41] Norman D. A. Bobrow D. G. (1975). On data-limited and resource-limited processes. Cogn. Psychol. 7, 44–64. doi: 10.1016/0010-0285(75)90004-3

[ref42] Nyhus E. Barceló F. (2009). The Wisconsin card sorting test and the cognitive assessment of prefrontal executive functions: a critical update. Brain Cogn. 71, 437–451. doi: 10.1016/j.bandc.2009.03.005, 19375839

[ref43] Olvet D. M. Hajcak G. (2009). The stability of error-related brain activity with increasing trials. Psychophysiology 46, 957–961. doi: 10.1111/j.1469-8986.2009.00848.x, 19558398

[ref44] Ota K. Shinya M. Kudo K. (2019). Transcranial direct current stimulation over dorsolateral prefrontal cortex modulates risk-attitude in motor decision-making. Front. Hum. Neurosci. 13:297. doi: 10.3389/fnhum.2019.00297, 31551733 PMC6743341

[ref45] Petruo V. A. Mückschel M. Beste C. (2018). On the role of the prefrontal cortex in fatigue effects on cognitive flexibility-a system neurophysiological approach. Sci. Rep. 8:6395. doi: 10.1038/s41598-018-24834-w, 29686384 PMC5913330

[ref46] Pinal D. Zurrón M. Díaz F. (2015). Age-related changes in brain activity are specific for high order cognitive processes during successful encoding of information in working memory. Front. Aging Neurosci. 7:75. doi: 10.3389/fnagi.2015.00075, 26029099 PMC4426757

[ref47] Rozand V. Lepers R. (2017). Influence de la fatigue mentale sur les performances physiques. Mov. Sport Sci. Sci. Mot. 95, 3–12.46. doi: 10.1051/sm/2015045

[ref48] Sally D. (2014). Numbers game-why everything you know about football is wrong. London, UK: Penguin Books Limited.

[ref49] Sarter M. Gehring W. J. Kozak R. (2006). More attention must be paid: the neurobiology of attentional effort. Brain Res. Rev. 51, 145–160. doi: 10.1016/j.brainresrev.2005.11.002, 16530842

[ref50] Schmit C. Brisswalter J. (2018). Executive functioning during prolonged exercise: a fatigue-based neurocognitive perspective. Int. Rev. Sport Exerc. Psychol. 13, 21–39. doi: 10.1080/1750984X.2018.1483527

[ref51] Schmitter-Edgecombe M. Langill M. (2006). Costs of a predictable switch between simple cognitive tasks following severe closed-head injury. Neuropsychology 20, 675–684. doi: 10.1037/0894-4105.20.6.675, 17100512 PMC1779821

[ref52] Skala F. Zemková E. (2022). Effects of acute fatigue on cognitive performance in team sport players: does it change the way they perform? A scoping review. Appl. Sci. 12:1736. doi: 10.3390/app12031736

[ref53] Smith M. R. Chai R. Nguyen H. T. Marcora S. M. Coutts A. J. (2019). Comparing the effects of three cognitive tasks on indicators of mental fatigue. J. Psychol. 153, 759–783. doi: 10.1080/00223980.2019.1611530, 31188721

[ref54] Smith M. R. Coutts A. J. Merlini M. Deprez D. Lenoir M. Marcora S. M. (2016). Mental fatigue impairs soccer-specific physical and technical performance. Med. Sci. Sports Exerc. 48, 267–276. doi: 10.1249/MSS.0000000000000762, 26312616

[ref55] Smith M. R. Marcora S. M. Coutts A. J. (2015). Mental fatigue impairs intermittent running performance. Med. Sci. Sports Exerc. 47, 1682–1690. doi: 10.1249/MSS.0000000000000592, 25494389

[ref56] Smith M. R. Thompson C. Marcora S. M. Skorski S. Meyer T. Coutts A. J. (2018). Mental fatigue and soccer: current knowledge and future directions. Sports Med. 48, 1525–1532. doi: 10.1007/s40279-018-0908-2, 29623604

[ref57] Smith M. R. Zeuwts L. Lenoir M. Hens N. De Jong L. M. Coutts A. J. (2016). Mental fatigue impairs soccer-specific decision-making skill. J. Sports Sci. 34, 1297–1304. doi: 10.1080/02640414.2016.1156241, 26949830

[ref58] Sokhadze E. M. Casanova M. F. Casanova E. L. Lamina E. Khachidze I. N. (2017). Event-related potentials (ERP) in cognitive neuroscience research and applications. Neuroregulation 4, 14–27. doi: 10.15540/nr.4.1.14

[ref59] Soylu Y. Ramazanoglu F. Arslan E. Clemente F. (2022). Effects of mental fatigue on the psychophysiological responses, kinematic profiles, and technical performance in different small-sided soccer games. Biol. Sport 39, 965–972. doi: 10.5114/biolsport.2022.110746, 36247954 PMC9536376

[ref60] Stroop J. R. (1935). Studies of interference in serial verbal reactions. J. Exp. Psychol. 18, 643–662. doi: 10.1037/h0054651

[ref61] Sun H. Soh K. G. Mohammadi A. Wang X. Bin Z. Zhao Z. (2022). Effects of mental fatigue on technical performance in soccer players: a systematic review with a meta-analysis. Front. Public Health 10:922630. doi: 10.3389/fpubh.2022.922630, 35937235 PMC9354787

[ref62] Sun H. Soh K. G. Roslan S. Wazir M. R. W. N. Soh K. L. (2021). Does mental fatigue affect skilled performance in athletes? A systematic review. PLoS One 16:e0258307. doi: 10.1371/journal.pone.0258307, 34648555 PMC8516214

[ref63] Thönes S. Falkenstein M. Gajewski P. D. (2018). Multitasking in aging: ERP correlates of dual-task costs in young versus low, intermediate, and high performing older adults. Neuropsychologia 119, 424–433. doi: 10.1016/j.neuropsychologia.2018.09.003, 30218690

[ref64] Tran Y. Craig A. Craig R. Chai R. Nguyen H. (2020). The influence of mental fatigue on brain activity: evidence from a systematic review with meta-analyses. Psychophysiology 57:e13554. doi: 10.1111/psyp.13554, 32108954

[ref65] Trecroci A. Boccolini G. Duca M. Formenti D. Alberti G. (2020). Mental fatigue impairs physical activity, technical and decision-making performance during small-sided games. PLoS One 15, e0238461–e0238412. doi: 10.1371/journal.pone.0238461, 32903263 PMC7480836

[ref66] Van Cutsem J. De Pauw K. Marcora S. Meeusen R. Roelands B. (2017). A caffeine-maltodextrin mouth rinse counters mental fatigue. Psychopharmacology 235, 947–958. doi: 10.1007/s00213-017-4809-0, 29247343

[ref67] Van der Linden D. Frese M. Meijman T. F. (2003). Mental fatigue and the control of cognitive processes: effects on perseveration and planning. Acta Psychol. 113, 45–65. doi: 10.1016/S0001-6918(02)00150-6, 12679043

[ref68] Verburgh L. Königs M. Scherder E. J. Oosterlaan J. (2014). Physical exercise and executive functions in preadolescent children, adolescents and young adults: a meta-analysis. Br. J. Sports Med. 48, 973–979. doi: 10.1136/bjsports-2012-091441, 23467962

[ref69] Vestberg T. Jafari R. Almeida R. Maurex L. Ingvar M. Petrovic P. (2020). Level of play and coach-rated game intelligence are related to performance on design fluency in elite soccer players. Sci. Rep. 10:9852. doi: 10.1038/s41598-020-66180-w, 32587269 PMC7316809

[ref70] Wallace J. L. Norton K. I. (2014). Evolution of world cup soccer final games 1966-2010: game structure, speed and play patterns. J. Sci. Med. Sport 17, 223–228. doi: 10.1016/j.jsams.2013.03.016, 23643671

[ref71] Wang Y. Yang Y. Xiao W. T. Su Q. (2016). Validity and reliability of the Chinese version of the cognitive flexibility inventory in a sample of college students. J. Ment. Health 30, 58–63. Available online at: https://www.cnki.net/

[ref72] Yuan R. Sun H. Soh K. G. Mohammadi A. Toumi Z. Zhang Z. (2023). The effects of mental fatigue on sport-specific motor performance among team sport athletes: a systematic scoping review. Front. Psychol. 14:1143618. doi: 10.3389/fpsyg.2023.1143618, 37113120 PMC10128192

[ref73] Zhou J. (2021). Differences on prosaccade task in skilled and less skilled female adolescent soccer players. Front. Psychol. 12:711420. doi: 10.3389/fpsyg.2021.711420, 34721156 PMC8551357

[ref74] Zühlsdorff K. Dalley J. W. Robbins T. W. Morein-Zamir S. (2023). Cognitive flexibility: neurobehavioral correlates of changing one's mind. Cereb. Cortex 33, 5436–5446. doi: 10.1093/cercor/n431 36368894 PMC10152092

